# Case Report: Compound heterozygous variants in *BHLHA9* cause complex syndactyly with oligodactyly, renal artery variation, and facial scar

**DOI:** 10.3389/fped.2025.1611387

**Published:** 2025-07-31

**Authors:** Weidong Wei, Xiaosha Wang, Tao Zhang, Yongxing Zhong, Jintang Zhang, Hua Yuan, Xiaoliang Shi, Yao He, Haitao Pan, Zhen Yang, Yuejuan Wang

**Affiliations:** ^1^ Shaoxing Maternity and Child Health Care Hospital, Shaoxing, Zhejiang, China; ^2^ Obstetrics and Gynecology Hospital of Shaoxing University, Shaoxing, Zhejiang, China; ^3^ Clinic Lab, BGI Genomics, Shanghai, China; ^4^ Shaoxing People’s Hospital, Shaoxing, Zhejiang, China

**Keywords:** compound heterozygosity, complex syndactyly, limb development, exome sequencing, *BHLHA9*

## Abstract

**Background:**

The *BHLHA9* gene, a member of the basic helix-loop-helix (bHLH) family of transcription factors, plays a critical role in limb development. Mutations in *BHLHA9* have been associated with various limb malformations, including syndactyly and split-hand/foot malformation. This study aimed to identify and characterize novel *BHLHA9* variants in a fetus with complex limb and renal abnormalities, providing further insights into the genetic basis of developmental disorders.

**Methods:**

We performed Exome sequencing (ES) on a fetus with severe limb malformations and renal anomalies, along with the parents. Sanger sequencing was used to validate the identified variants. Evolutionary conservation analysis and structural predictions using AlphaFold were conducted to assess the functional impact of the variants. Protein-protein interaction networks were generated using the STRING database to explore potential functional partners of BHLHA9.

**Results:**

The proband exhibited multicystic dysplasia of the left kidney, an accessory renal artery, bilateral hand anomalies (four fingers with absent thumbs), bilateral foot syndactyly, and a facial scar. ES identified two novel compound heterozygous variants in the *BHLHA9* gene: c.251C>T (p.Ala84Val) inherited from the father, and c.250_261dup (p.Ala84_Ala87dup) inherited from the mother. The two variants all located within the helix-loop-helix (HLH) domain, a critical region for protein-protein interactions and DNA binding. Evolutionary conservation analysis revealed that the affected residues are highly conserved across species, and structural predictions suggested that the two variants may disrupt the HLH domain's structural integrity. Protein-protein interaction analysis identified several potential functional partners of BHLHA9, including ASCL5, YWHAE, and PAFAH1B1, which are involved in transcriptional regulation, signaling pathways, and neuronal migration, respectively.

**Conclusions:**

This study identifies novel compound heterozygous variants in the *BHLHA9* gene represents a rare autosomal recessive disorder with severe limb and renal abnormalities. The c.251C>T and c.250_261dup variants, located within the HLH domain, is predicted to impair protein function, potentially disrupting limb development. These findings expand the spectrum of *BHLHA9* mutations linked to developmental disorders and highlight the importance of the HLH domain in BHLHA9's regulatory role.

## 1 Introduction

Limb development during embryogenesis is a highly intricate and tightly regulated process, involving the coordinated action of numerous genes, signaling pathways, and transcriptional regulators. Key genes such as *HOXD13*(OMIM#142989), *GLI3*(OMIM#165240), *SHH* (OMIM#600725), *FGF8*(OMIM#600483), *WNT7A* (OMIM#601570), and *WNT10B* (OMIM#601906) play critical roles in early limb patterning and morphogenesis (1–3). Disruptions in these molecular networks can lead to a spectrum of congenital limb malformations, which are clinically and genetically heterogeneous. Among these, mesoaxial synostotic syndactyly with phalangeal reduction (MSSD, OMIM#609432) represents a rare autosomal recessive disorder characterized by mesoaxial osseous fusion at the metacarpal level, reduction in the number of phalanges, hypoplasia of the distal phalanges, clinodactyly of the fifth fingers, and preaxial fusion of the toes (4). MSSD is one of the few nonsyndromic syndactylies inherited in a recessive manner, highlighting its unique genetic etiology

Recent advances in genetic studies have identified *BHLHA9* (Basic Helix-Loop-Helix Family Member A9, OMIM#615416), located on chromosome 17p13.3, as a pivotal gene in limb development (5, 6). *BHLHA9* encodes a transcription factor belonging to the basic helix-loop-helix (bHLH) family, which is exclusively expressed in the distal mesenchyme of limb buds during mouse embryonic stages E10.5-E11.5. This spatiotemporal expression pattern underscores its role in the regulation of limb morphogenesis, particularly in the patterning of digital rays. Functional studies have demonstrated that BHLHA9 acts as a master regulator, modulating the transcriptional network essential for normal limb development (7). Alterations in BHLHA9 dosage or function disrupt the signaling interactions between the apical ectodermal ridge (AER) and the progress zone (PZ), leading to limb malformations.

To date, *BHLHA9* has been implicated in three distinct limb phenotypes: split-hand/foot malformation with long bone deficiency (SHFLD3, OMIM#612576), complex camptosynpolydactyly (OMIM#607539), and MSSD. Heterozygous duplications or triplications of *BHLHA9* are associated with SHFLD3, an autosomal dominant condition characterized by variable expressivity and incomplete penetrance (5). In contrast, homozygous loss-of-function mutations in *BHLHA9*, including missense and frameshift variants, have been linked to MSSD and complex camptosynpolydactyly (4, 8). Notably, all reported MSSD cases with *BHLHA9* mutations have originated from consanguineous families, predominantly of Pakistani descent, suggesting a founder effect or a high prevalence of these mutations in certain populations.

In this study, we report compound heterozygous variants in *BHLHA9*, c.250_261dup (p.Ala84_Ala87dup) and c.251C>T (p.Ala84Val), as the cause of a novel case of complex syndactyly. These variants, located in a critical region of the protein, likely disrupt BHLHA9's structural and functional integrity, expanding the mutational and phenotypic spectrum of *BHLHA9*-related limb malformations. This finding underscores the importance of compound heterozygosity in the pathogenesis of complex syndactyly and further establishes *BHLHA9* as a key regulator of limb development.

## 2 Materials and methods

### 2.1 Sample collection

Fetal samples were obtained from abortion tissues, while venous blood samples (5 ml) were collected from the parents. Written informed consent was provided by the parents prior to participation. The study protocol was reviewed and approved by the Ethics Committee of Shaoxing Maternal and Child Health Care Hospital

### 2.2 Exome sequencing

Exome sequencing (ES) was performed on the proband and his parents. Genomic DNA was isolated from venous blood samples using the DNEasy Blood and Tissue Kit (Qiagen, Hilden, Germany), following the manufacturer's protocol. Coding exons were captured using the Agilent SureSelect Low Input Reagent Kit, and sequencing was carried out on the Illumina HiSeq X Ten platform. The sequencing data for the proband covered 99.89% of the coding regions, spanning a total of 58,682,415 bp across 25,701 genes. The sequencing achieved an average depth of 263.37× across target regions, with 98.75% of bases covered at ≥20× depth.

### 2.3 Data processing and variant analysis

Raw sequencing data underwent quality control using FastQC (v0.12.1) and Trimmomatic (v0.39) to remove low-quality reads (Phred score <20, length <50 bp) and adapter sequences (9). Clean reads were aligned to the GRCh37/hg19 reference genome via BWA-MEM (v0.7.17) (10), followed by GATK (v4.2.6.1)-based variant calling for SNVs and indels. Variants were filtered using strict thresholds: allele frequency <0.1% in gnomAD v2.1.1 and <1% in 1000 Genomes, functional impact prioritization (missense/nonsense/splice-site variants), and quality metrics (depth ≥10×, GQ ≥20). Annotation leveraged ClinVar, HGMD Pro, and OMIM for clinical relevance, supplemented by dbNSFP v4.3a (SIFT, PolyPhen-2, CADD) for pathogenicity prediction. ACMG/AMP classification was performed using InterVar (v2.2.3) with manual curation of population data (PM2), computational evidence (PP3/BP4), and segregation analysis. Candidate variants were validated via Sanger sequencing on an ABI 3730xl system (BigDye v3.1).

### 2.4 Sanger sequencing validation

Candidate variants were validated by Sanger sequencing in the proband and parents. PCR amplification was performed using standard reaction conditions: 35 cycles with annealing at 58°C. Purified PCR products were sequenced on an ABI 3730xl Genetic Analyzer (Thermo Fisher Scientific) using BigDye Terminator v3.1 chemistry. Electropherograms were analyzed with SeqMan Pro (DNASTAR Lasergene 17) to confirm variant segregation.

### 2.5 Protein evolutionary conservation and structural analysis

To assess the evolutionary conservation of the BHLHA9 protein, we utilized the SMART (Simple Modular Architecture Research Tool) database (available at http://smart.embl-heidelberg.de/). The amino acid sequences of BHLHA9 from multiple species, including Homo sapiens, rat, mouse, zebrafish, and fruit fly, were aligned to identify conserved regions, particularly within the helix-loop-helix (HLH) domain. For structural analysis of the BHLHA9 protein and its interaction partners, we employed the STRING database (available at https://cn.string-db.org/). The STRING tool was used to predict protein-protein interaction networks based on known and predicted interactions, including physical and functional associations.

## 3 Results

### 3.1 Clinical features

**Figure 1:**
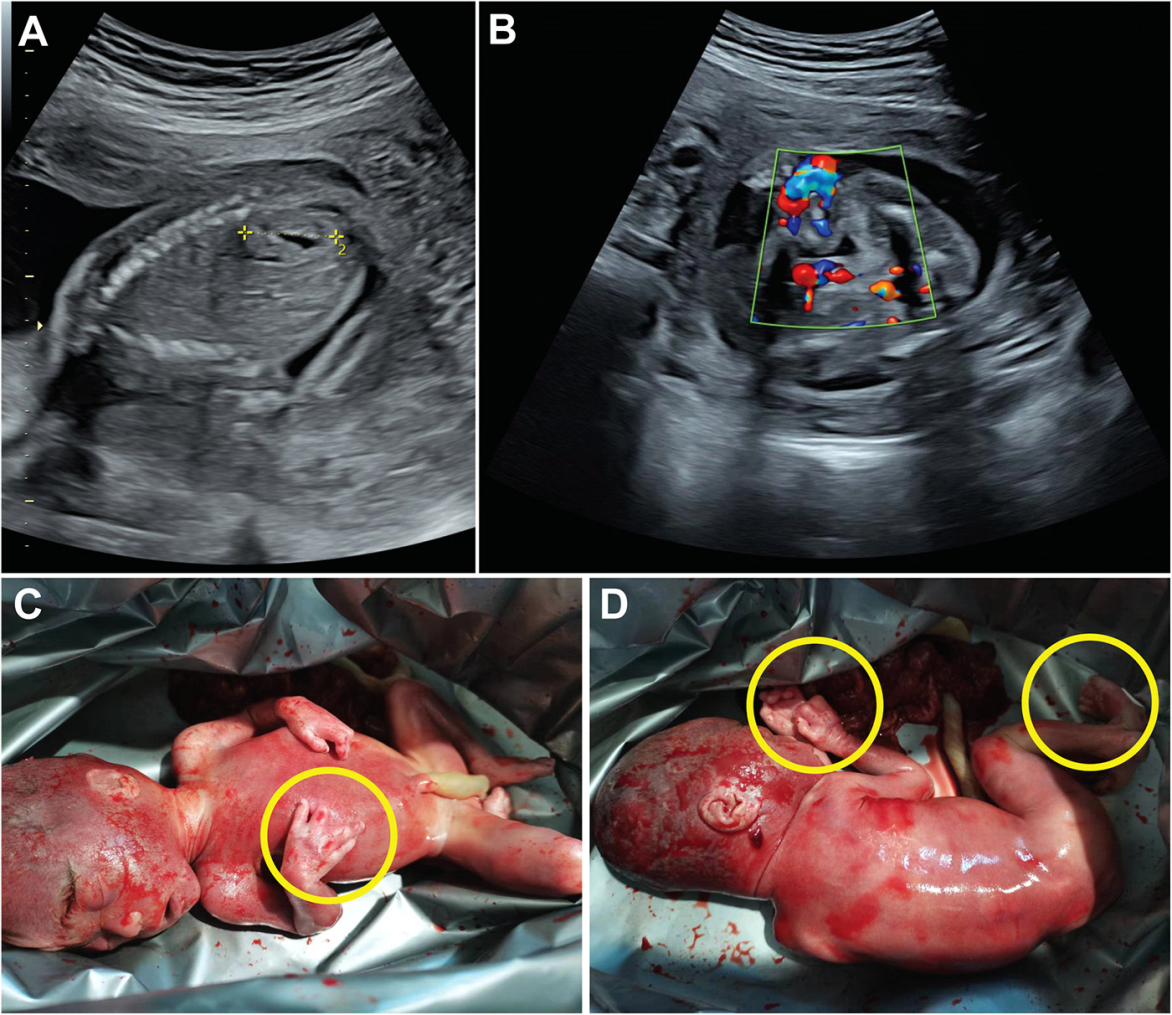
Phenotypic features of the fetus following termination of pregnancy. Ultrasound images labeled **A** and **B** show prenatal scans. Images **C** and **D** depict a newborn with yellow circles marking limb abnormalities.

The proband was a fetus at 23 weeks of gestation. Prenatal ultrasound revealed multicystic dysplasia of the left kidney and an accessory renal artery in the right kidney (Figures 1A,B). Following termination of pregnancy, postmortem examination identified additional phenotypic abnormalities, including bilateral hand anomalies (four fingers on each hand with absent thumbs), bilateral foot syndactyly, and a facial scar (Figures 1C,D). During the pregnancy, the mother reported taking ibuprofen once during weeks 3–4 for a cold and contracting influenza A during week 10 without medication. Non-invasive prenatal testing (NIPT) and nuchal translucency (NT) screening showed no significant abnormalities. There was no notable family history of congenital anomalies or genetic disorders.

### 3.2 Identification of *BHLHA9* variants in the family

**Figure 2:**
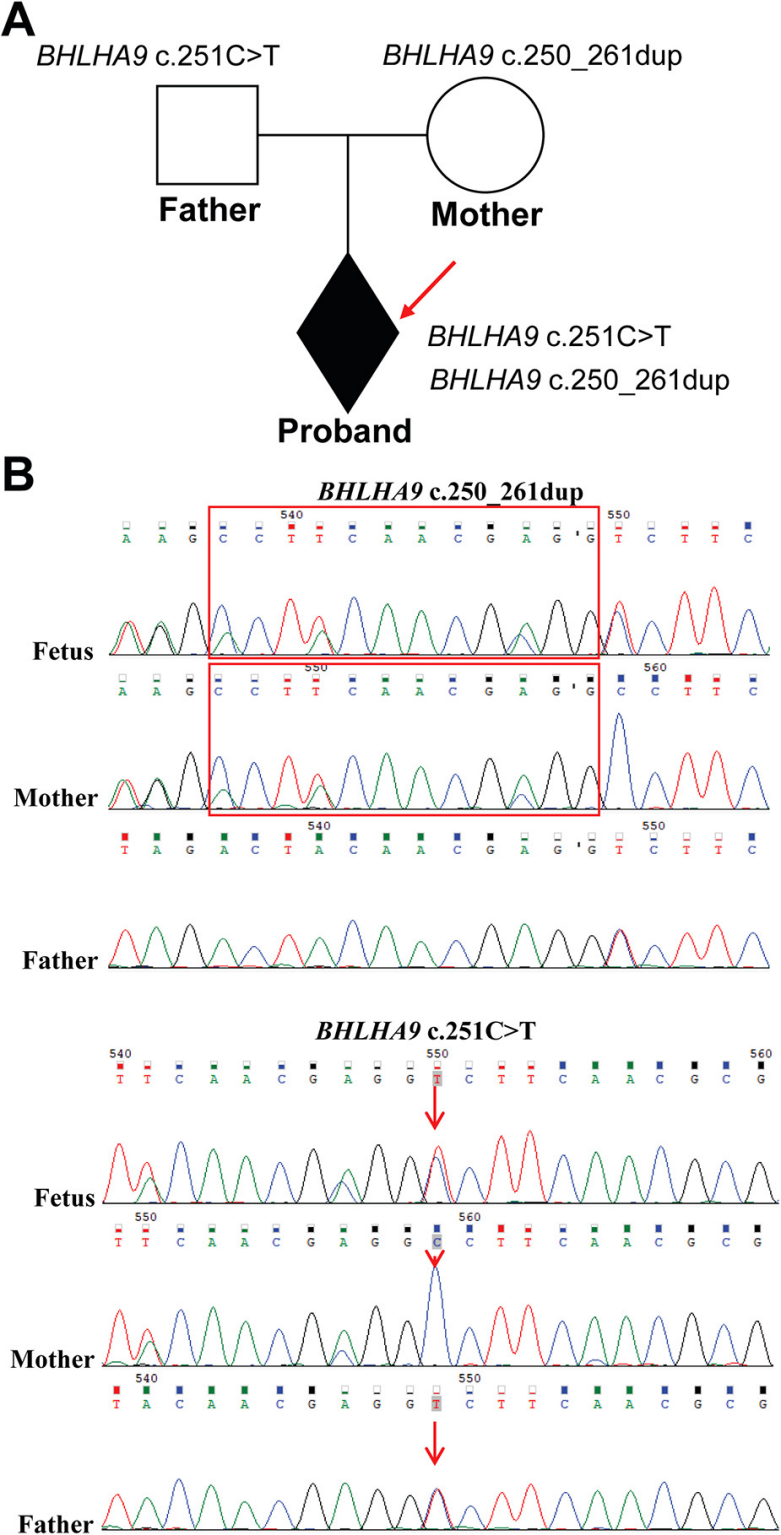
Identification of *BHLHA9* variants in the family. **(A)** The family pedigree illustrates the inheritance pattern, with the proband indicated by an arrow. **(B)** ES and Sanger sequencing revealed two variants in the *BHLHA9* gene in the proband. The c.251C>T (p.Ala84Val) variant was inherited from the father, while the c.250_261dup (p.Ala84_Ala87dup) variant inherited from the mother, resulting in a compound heterozygous state in the proband. The red box highlights the repetitive area (c.250_261dup).

To investigate the genetic basis of the observed phenotypic abnormalities, we performed Exome sequencing (ES) on the proband and his parents. ES detected two previously unreported compound heterozygous variants in the *BHLHA9* gene. The *BHLHA9* NM_001164405.2 c.251C>T (p.Ala84Val) variant was inherited from the father, while the *BHLHA9* NM_001164405.2 c.250_261dup (p.Ala84_Ala87dup) variant was maternally inherited, which resulting in a compound heterozygous state in the proband (Figure 2A). Sanger sequencing validation confirmed the presence of both variants in the proband and their respective parental origins (Figure 2B). The c.251C>T (p.Ala84Val) variant results in a missense mutation, while the c.250_261dup (p.Ala84_Ala87dup) variant leads to an in-frame duplication within the helix-loop-helix (HLH) domain of the BHLHA9 protein. The c.251C>T (p.Ala84Val) variant is classified as uncertain significance in ClinVar. Meanwhile, the c.250_261dup (p.Ala84_Ala87dup) variant has no record in the database.

### 3.3 ClinVar-reported variants in the *BHLHA9* gene

**Figure 3:**
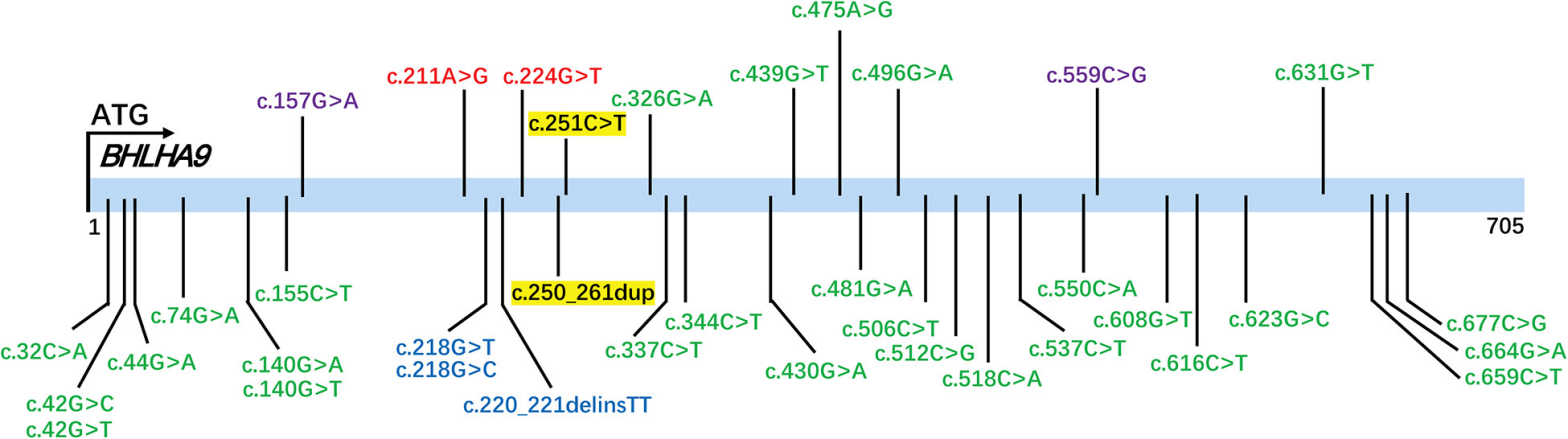
ClinVar-Reported variants in the *BHLHA9* gene. The figure displays variants in the *BHLHA9* gene documented in the ClinVar database, color-coded based on their classification: green for Variants of Uncertain Significance (VUS), red for pathogenic (P), and blue for likely pathogenic (LP). Variants highlighted in yellow represent those identified and reported in the current study.

To contextualize the variants identified in our study, we analyzed the *BHLHA9* gene variants documented in the ClinVar database (Figure 3). Variants highlighted in yellow represent those identified and reported in the current study, including c.251C>T (p.Ala84Val) and c.250_261dup (p.Ala84_Ala87dup). The ClinVar-reported variants span multiple regions of the *BHLHA9* gene, with several located within the helix-loop-helix (HLH) domain, a critical functional region for protein-protein interactions and DNA binding. Notably, the c.250_261dup variant identified in our study falls within this domain, suggesting potential functional consequences.

We classified the variants according to the ACMG/AMP guidelines. The c.250_261dup (p.Ala84_Ala87dup) in-frame duplication was absent in population databases (1000 Genomes, ExAC, and gnomAD; PM2_Supporting) and predicted to disrupt protein structure via a 4-alanine repeat (PM4). Given limited evidence, it was classified as Uncertain Significance (VUS). For the c.251C>T (p.Ala84Val) missense variant, its extremely low gnomAD frequency (0.00009; PM2_Supporting) and concordant computational evidence strongly supported pathogenicity: SIFT (“Affect protein function”), REVEL (0.872; pathogenic threshold >0.75), VEST4 (0.865; >0.8), BayesDel_noAF (0.268677; >0.2), and MetaSVM (17.5/20).

### 3.4 Functional and structural implications of *BHLHA9* variants in the HLH domain

**Figure 4:**
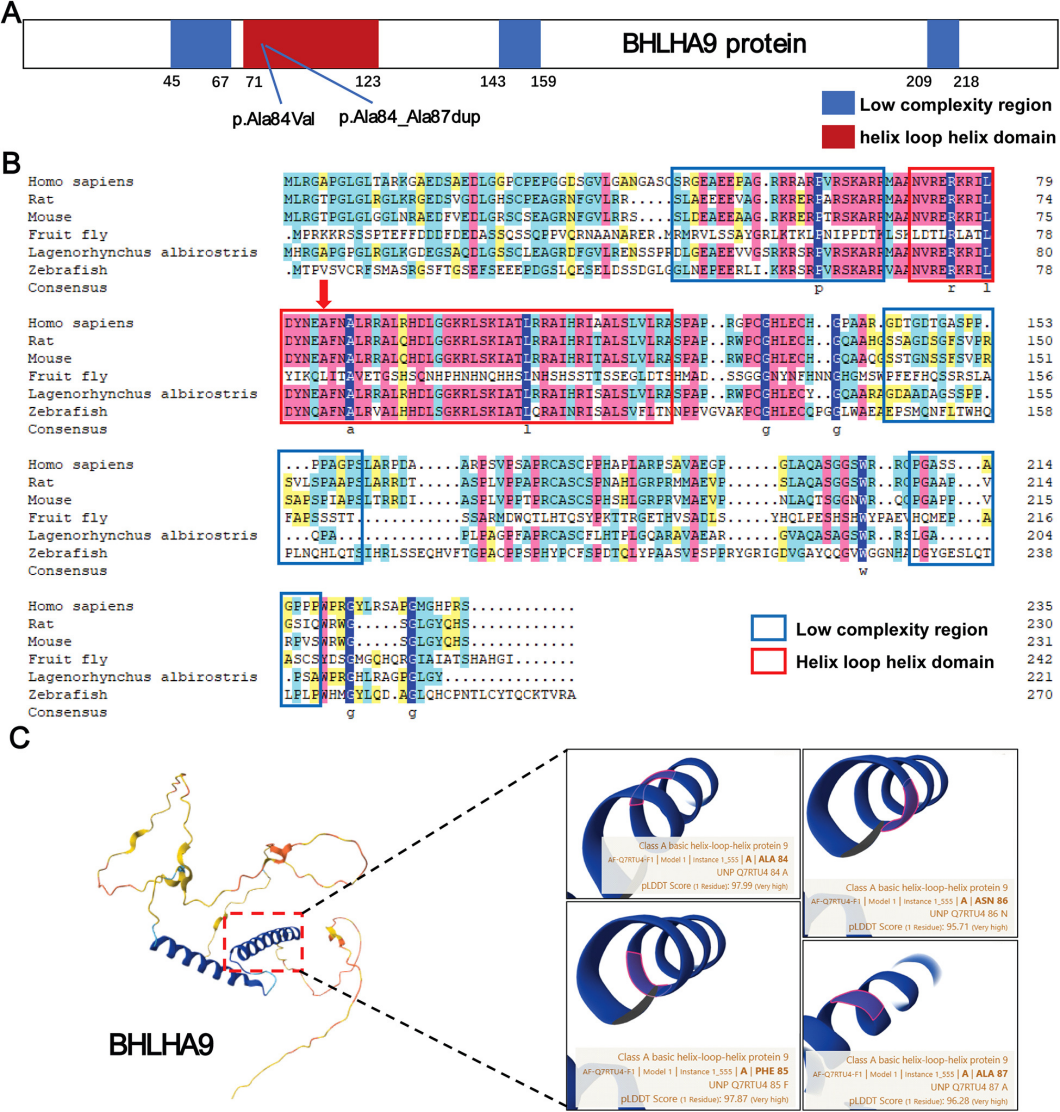
Structural and functional analysis of BHLHA9 protein variants. **(A)** Schematic representation of the BHLHA9 protein, highlighting key domains and the locations of identified variants. The p.Ala84Val variant and p.Ala84_Ala87dup variant are located in the helix-loop-helix (HLH) domain, a critical functional region of the protein. **(B)** Sequence alignment of BHLHA9 across multiple species (Homo sapiens, rat, mouse, fruit fly, Lagenorhynchus albirostris, and zebrafish), demonstrating evolutionary conservation of the affected regions. Colors are used to represent the conservation levels of amino acids. Dark blue highlights highly conserved amino acids across all species depicted, while lighter shades indicate less conserved ones. **(C)** Structural analysis of BHLHA9 variants using AlphaFold, illustrating the predicted impact of the p.Ala84Val and p.Ala84_Ala87dup variants on protein conformation.

In this study, we identified two variants in the *BHLHA9* gene, p.Ala84Val and p.Ala84_Ala87dup, both located within the helix-loop-helix (HLH) domain, a critical region for protein-protein interactions and DNA binding. The p.Ala84Val variant is situated within the HLH domain, while the p.Ala84_Ala87dup variant involves a duplication within the HLH domain, potentially altering its structural and functional properties (Figure 4A). Evolutionary conservation analysis across multiple species (Homo sapiens, rat, mouse, fruit fly, Lagenorhynchus albirostris, and zebrafish) demonstrated that the affected residues in the HLH domain are highly conserved, underscoring their functional importance. This conservation suggests that perturbations in these regions, such as those caused by the identified variants, could have significant biological consequences (Figure 4B). Structural analysis using AlphaFold further elucidated the potential impact of these variants. Both p.Ala84Val and p.Ala84_Ala87dup are predicted to disrupt the structural integrity of the HLH domain, which is crucial for protein-protein interactions and DNA binding. The p.Ala84_Ala87dup variant, in particular, may induce conformational changes that impair the domain's functionality (Figure 4C).

### 3.5 Predicted functional partners of *BHLHA9*

**Figure 5:**
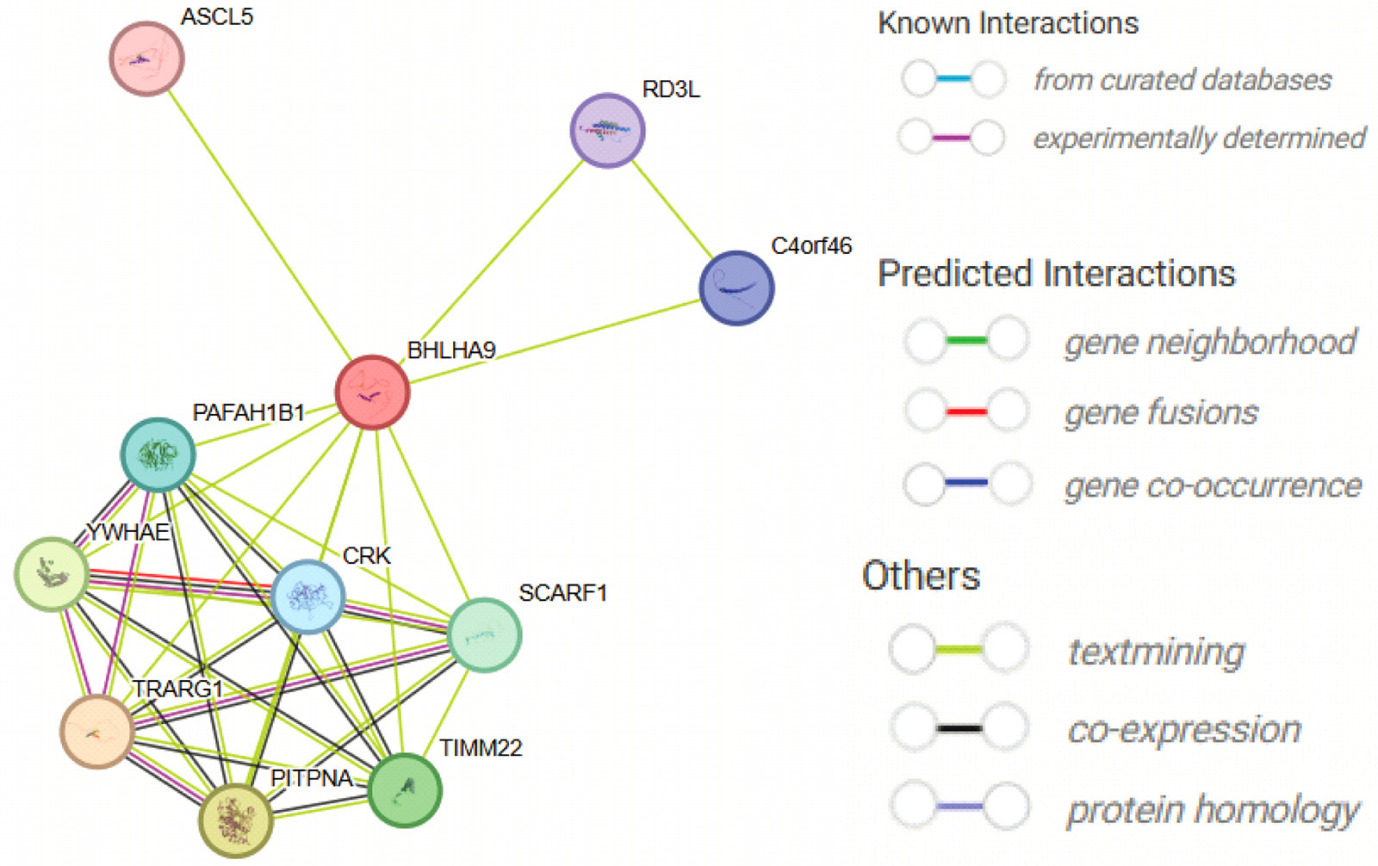
Protein-Protein interaction network of BHLHA9 predicted by STRING. The figure illustrates the predicted protein-protein interaction network of BHLHA9, generated using the STRING database. Key interacting partners include ASCL5, RD3l, C4orf46, TRARG1, PAFAH1B1, WHAEL, CRK, SCARF1, TIMM22, and PITFNA. These interactions suggest potential functional associations of BHLHA9 in various biological processes, such as transcriptional regulation, signaling pathways, and cellular metabolism. The network highlights the central role of BHLHA9 and its potential involvement in complex protein interactions, providing insights into its biological functions and regulatory mechanisms.

To preliminarily explore the potential biological roles of BHLHA9, we performed in silico protein-protein interaction (PPI) analysis using the STRING database, which predicts functional associations based on co-expression, phylogenetic profiling, and text-mining data. The generated network suggested potential interactions with proteins including ASCL5, RD3l, C4orf46, TRARG1, PAFAH1B1, YWHAE, CRK, SCARF1, TIMM22, and PITPNA (Figure 5). It is important to note that these interactions remain speculative and require experimental validation. Functional annotation of the predicted partners (Supplementary Table S1) indicated their hypothetical roles in transcriptional regulation (ASCL5), signaling pathways (YWHAE, CRK), and metabolic processes (TRARG1).

## 4 Discussion

The *BHLHA9* gene, a member of the basic helix-loop-helix (bHLH) family of transcription factors, plays a critical role in limb development. The bHLH proteins are highly conserved across species and are involved in various developmental processes, including limb morphogenesis, muscle development, and nervous system formation (8, 11). *BHLHA9*, located on chromosome 17p13.3, has been implicated in limb malformations, particularly through its role in regulating gene expression during early embryonic development. Our study identified two novel variants in *BHLHA9*, c.251C>T (p.Ala84Val) and c.250_261dup (p.Ala84_Ala87dup), both located within the HLH domain, which is essential for protein-protein interactions and DNA binding. These findings expand the spectrum of *BHLHA9* mutations associated with limb developmental disorders and provide further insights into the molecular mechanisms underlying these phenotypes.

The HLH domain of BHLHA9 is crucial for dimerization with other bHLH proteins, which is necessary for its transcriptional regulatory function. In our study, we identified compound heterozygous variants within the HLH domain of BHLHA9, including the c.250_261dup variant. Both variants are located in the critical HLH domain, which is essential for dimerization with other bHLH proteins. The c.250_261dup variant, in particular, is predicted to disrupt the alpha-helical structure of the HLH domain, likely impairing its ability to form functional dimers and execute its transcriptional regulatory functions. This finding is consistent with the severe limb malformations observed in our case, including oligodactyly and syndactyly, further underscoring the importance of the HLH domain in BHLHA9-mediated limb development. This is consistent with previous studies showing that mutations in the HLH domain of bHLH proteins can lead to severe developmental defects. Klopocki et al. demonstrated that knockdown of *bhlha9* in zebrafish resulted in pectoral fin shortening, highlighting its role in limb development (7). Similarly, Schatz et al. observed that homozygous *bhlha9* knockout mice exhibited syndactyly, further supporting the importance of *BHLHA9* in limb morphogenesis (12).

Our findings are also consistent with reports of *BHLHA9* mutations in human limb malformations. Phadke et al. identified a pathogenic mutation in *BHLHA9* associated with complex camptosynpolydactyly, characterized by severe hand and foot abnormalities (13). Additionally, Malik et al. reported that missense mutations in the DNA-binding region of *BHLHA9* are associated with mesoaxial synostotic syndactyly (MSSD), a condition characterized by fusion of the middle digits (4). These studies, along with our results, underscore the critical role of *BHLHA9* in limb development and suggest that mutations in this gene can lead to a spectrum of limb malformations.

The evolutionary conservation of the HLH domain further supports the functional importance of BHLHA9. Our analysis revealed that the amino acids affected by the identified variants are highly conserved across species, suggesting that perturbations in this region are likely to have significant biological consequences. Structural predictions using AlphaFold indicated that the c.250_261dup variant may induce conformational changes in the HLH domain, potentially disrupting its interaction with other proteins and impairing its regulatory function. This is consistent with the observed phenotypic abnormalities in the proband, including limb malformations and renal anomalies. The renal anomalies, including multicystic dysplasia and accessory renal artery, may arise from disrupted BHLHA9-dependent networks. Key interaction partners of BHLHA9, such as YWHAE and PAFAH1B1, are directly implicated in renal development. YWHAE regulates WNT/β-catenin signaling in nephron progenitor cells (14). Furthermore, Chouery reported renal anomalies in a family with *BHLHA9* duplications, supporting a pleiotropic role of *BHLHA9* in multi-organ development (15). The compound heterozygous variants identified here likely impair these pathways, leading to concurrent limb and renal defects.

The phenotypic variability observed in individuals with *BHLHA9* mutations may be influenced by genetic modifiers or environmental factors. Duplications or triplications of the *BHLHA9* gene have been associated with split-hand/foot malformation with long bone deficiency (SHFLD3), a condition characterized by variable expressivity and incomplete penetrance (16, 17). This suggests that the dosage of *BHLHA9* may play a critical role in limb development, with increased copy number leading to more severe phenotypes. However, the exact mechanisms underlying this dosage effect remain to be fully understood. In addition to its role in limb development, BHLHA9 may interact with other proteins involved in signaling pathways critical for embryonic development. Our protein-protein interaction analysis identified several potential partners, including ASCL5, YWHAE, and PAFAH1B1, which are involved in transcriptional regulation, signaling pathways, and neuronal migration, respectively. These interactions suggest that BHLHA9 may participate in a broader regulatory network, influencing multiple aspects of development, including limb and renal morphogenesis. Further studies are needed to validate these interactions and elucidate their functional significance.

## 5 Conclusion

Our study highlights the importance of *BHLHA9* in limb development and provides further evidence that mutations in this gene can lead to a range of limb malformations. The identification of novel variants in the HLH domain expands our understanding of the genetic basis of these disorders and underscores the need for further functional studies to elucidate the precise mechanisms by which these mutations disrupt BHLHA9 function. These findings have important implications for the genetic diagnosis and counseling of families affected by limb malformations.

## Data Availability

The datasets presented in this study can be found in online repositories. The names of the repository/repositories and accession number(s) can be found in the article/Supplementary Material.
