# Supplementary material for: Case Report: Compound heterozygous variants in BHLHA9 cause complex syndactyly with oligodactyly, renal artery variation, and facial scar
Source: Front Pediatr. 2025 Jul 31;13:1611387. doi: 10.3389/fped.2025.1611387 (PMC12350244; doi:10.3389/fped.2025.1611387)
Supplement: Supplementary file 1 [file Table1.docx]

**Table S1. Predicted functional partners of BHLHA9 and their related protein functions**

| **Predicted Functional Partners** | **Related protein function** |
| --- | --- |
| BHLHA9 | Transcription factor, which play a role in limb development. Is an essential player in the regulatory network governing transcription of genes implicated in limb morphogenesis |
| TRARG1 | Regulates insulin-mediated adipose tissue glucose uptake and transport by modulation of SLC2A4 recycling. Not required for SLC2A4 membrane fusion upon an initial stimulus, but rather is necessary for proper protein recycling during prolonged insulin stimulation. |
| PITPNA | Catalyzes the transfer of PtdIns and phosphatidylcholine between membranes. |
| YWHAE | Adapter protein implicated in the regulation of a large spectrum of both general and specialized signaling pathways. Binds to a large number of partners, usually by recognition of a phosphoserine or phosphothreonine motif. Binding generally results in the modulation of the activity of the binding partner (By similarity). Positively regulates phosphorylated protein HSF1 nuclear export to the cytoplasm. |
| TIMM22 | Essential core component of the TIM22 complex, a complex that mediates the import and insertion of multi-pass transmembrane proteins into the mitochondrial inner membrane. In the TIM22 complex, it constitutes the voltage-activated and signal-gated channel. Forms a twin-pore translocase that uses the membrane potential as external driving force in 2 voltage-dependent steps. |
| SCARF1 | Mediates the binding and degradation of acetylated low-density lipoprotein (Ac-LDL). Mediates heterophilic interactions, suggesting a function as adhesion protein. Plays a role in the regulation of neurite-like outgrowth. |
| PAFAH1B1 | Required for proper activation of Rho GTPases and actin polymerization at the leading edge of locomoting cerebellar neurons and postmigratory hippocampal neurons in response to calcium influx triggered via NMDA receptors. Non-catalytic subunit of an acetylhydrolase complex which inactivates platelet-activating factor (PAF) by removing the acetyl group at the SN-2 position (By similarity). Positively regulates the activity of the minus-end directed microtubule motor protein dynein. |
| CRK | Regulates cell adhesion, spreading and migration. Mediates attachment-induced MAPK8 activation, membrane ruffling and cell motility in a Rac-dependent manner. Involved in phagocytosis of apoptotic cells and cell motility via its interaction with DOCK1 and DOCK4. May regulate the EFNA5-EPHA3 signaling. |
| C4orf46 | Chromosome 4 open reading frame 46. |
| RD3L | Retinal degeneration 3 like. |
| ASCL5 | Achaete-scute family bHLH transcription factor 5 |
